# From fat storage to immune hubs: the emerging role of adipocytes in coordinating the immune response to infection

**DOI:** 10.1111/febs.17302

**Published:** 2024-10-20

**Authors:** Matthew C. Sinton, Shingo Kajimura

**Affiliations:** ^1^ Division of Immunology, Immunity to Infection and Respiratory Medicine University of Manchester UK; ^2^ Lydia Becker Institute of Immunology and Inflammation University of Manchester UK; ^3^ Division of Endocrinology, Diabetes and Metabolism Beth Israel Deaconess Medical Center and Harvard Medical School Boston MA USA; ^4^ Howard Hughes Medical Institute Chevy Chase MD USA

**Keywords:** adipocytes, adipose tissue immunity, adipose‐immune communication, host–pathogen interactions, immune hubs

## Abstract

Adipose tissue is a rich source of diverse cell populations, including immune cells, adipocytes and stromal cells. Interactions between these different cell types are now appreciated to be critical for maintaining tissue structure and function, by governing processes such as adipogenesis, lipolysis and differentiation of white to beige adipocytes. Interactions between these cells also drive inflammation in obesity, leading to an expansion of adipose tissue immune cells, and the secretion of proinflammatory cytokines from immune cells and from adipocytes themselves. However, in evolutionary terms, obesity is a recent phenomenon, raising the question of why adipocytes evolved to express factors that influence the immune response. Studies of various pathogens indicate that adipocytes are highly responsive to infection, altering their metabolic profiles in a way that can be used to release nutrients and fuel the immune response. In the case of infection with the extracellular parasite *Trypanosoma brucei*, attenuating the ability of adipocytes to sense the cytokine IL‐17 results in a loss of control of the local immune response and an increased pathogen load. Intriguingly, comparisons of the adipocyte response to infection suggest that the immune responses of these cells occur in a pathogen‐dependent manner, further confirming their complexity. Here, with a focus on murine adipose tissue, we discuss the emerging concept that, in addition to their canonical function, adipocytes are immune signalling hubs that integrate and disseminate signals from the immune system to generate a local environment conducive to pathogen clearance.

## Graphical abstract

Traditionally, adipocytes have been viewed as energy storage units, which upregulate inflammatory factors in response to obesity. However, emerging evidence demonstrates that adipocytes are highly responsive to systemic infection, and upregulate cytokines and antimicrobial compounds in response. Here, we propose that adipocytes form immune hubs that are able to integrate local immune signals and, in turn, disseminate their own signals to control the local immune response and pathogen burden.

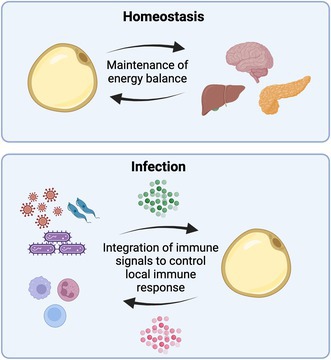

**Abbreviations**
AdipoR1: adiponectin receptor 1
ATGL: adipose triglyceride lipase
BAT: brown adipose tissue
Breg: B regulatory cell
C3: complement component 3
CCL12: chemokine (C‐C motif) ligand 12
Ccl6: chemokine (C‐C motif) ligand 6
Ccl8: chemokine (C‐C motif) ligand 8
Ccl9: chemokine (C‐C motif) ligand 9
CD4: cluster of differentiation 4
CD8: cluster of differentiation 8
cDC1: conventional dendritic cell type 1
cDC2: conventional dendritic cell type 2
CXCL10: C‐X‐C motif chemokine 10
Cxcl12: C‐X‐C motif chemokine 12
FFA: free fatty acid
GDF15: growth/differentiation factor 15
gWAT: gonadal white adipose tissue
HIV: human immunodeficiency virus
HSL: hormone‐sensitive lipase
IAV: influenza A virus
IFNγ: interferon gamma
IL‐10: interleukin 10
IL‐12: interleukin 12
IL‐13: interleukin 13
IL‐17A: interleukin 17A
IL‐17F: interleukin 17F
IL‐17RA: interleukin 17 receptor A
IL‐17RC: interleukin 17 receptor C
IL‐1β: interleukin 1B
IL‐22: interleukin 22
IL‐23: interleukin 23
IL‐33: interleukin 33
IL‐35: interleukin 35
IL‐4: interleukin 4
IL‐5: interleukin 5
IL‐6: interleukin 6
IL‐8: interleukin 8
ILC: innate lymphoid cell
ILC2: group 2 innate lymphoid cell
iNKT: invariant natural killer cell
LPS: lipopolysaccharide
SARS‐CoV‐2: severe acute respiratory syndrome‐related coronavirus 2
scWAT: subcutaneous white adipose tissue
TGF‐β: transforming growth factor beta
TGF‐β1: transforming growth factor beta 1
T_H_17: T helper 17 cell
T_H_2: T helper 2 cell
TLR2: toll‐like receptor 2
TLR4: toll‐like receptor 4
TLR9: toll‐like receptor 9
TNFα: tumour necrosis factor alpha
Treg: T regulatory cell
Ucp1: uncoupling protein 1
WAT: white adipose tissue

## Introduction

The adipose tissue, which was once thought of as an inert energy storage facility, is now understood to exert profound effects on energy balance, through both endocrine signalling and regulation of organismal behaviour. Whilst it is made up of a number of cell types, including stromal (e.g. fibroblasts and preadipocytes) and immune cells (e.g. macrophages), the major cell of the adipose tissue is the adipocyte, which, under physiological conditions, stores fuel, mainly in the form of triglycerides, which can be hydrolyzed through lipolysis and then released to fuel distal tissues. Advances in single‐cell transcriptomic studies have also revealed that there is heterogeneity within the adipocyte populations, with variation in these cell types across different white adipose tissue (WAT) depots [1, 2, 3]. Spatial transcriptomic analyses of human subcutaneous WAT (scWAT) also demonstrated that only a subset of adipocytes respond to insulin stimulation, and that there is a correlation between the size of this subset and whole‐body insulin sensitivity. Moreover, this study used neighbourhood analyses to reveal homotypic clustering of a separate adipocyte subset, with putative pro‐inflammatory properties [4]. These studies clearly highlight the previously unappreciated complexity and organisation of this tissue, and the need to understand the function of different subpopulations of adipocytes.

**Fig. 1:**
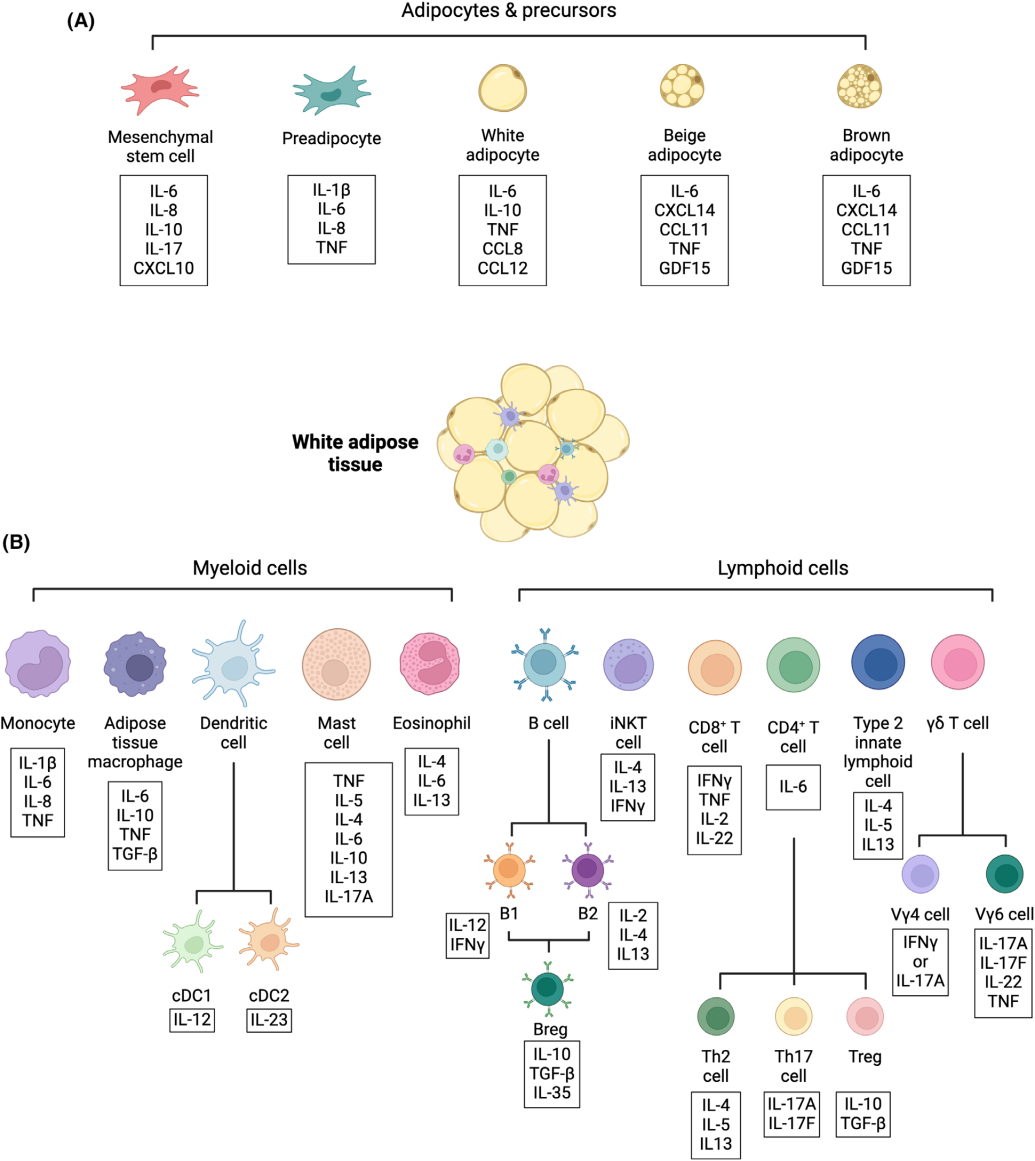
Immune composition of adipose tissue. (A) Schematic view of adipocyte subsets and precursors, and common cytokines that they express. (B) Schematic view of the multitude of immune cells within the white adipose tissue, and common cytokines that they express. The adipose tissue comprises ~ 30% adipocytes, with the remaining cells composed of stromal and immune cells. The adipocytes and their precursors (mesenchymal stem cells and preadipocytes) secrete cytokines under homeostatic conditions, which can play a role in controlling adipose tissue structure and function. There is also a classical view that the majority of immune cells within the adipose tissue are macrophages, but we now understand that the diversity of immune cells within this tissue is vast, and complex, as illustrated here.

Beyond their energy storage function, adipocytes also secrete bioactive proteins called adipokines, which regulate a range of physiological functions, including food intake [5], fat distribution [6, 7], energy expenditure [8] and body temperature [9]. In addition to their physiological roles, adipokines such as leptin play a role in the immune response, by stimulating immune cell activation and promoting inflammation [10]. The term adipokine also encompasses molecules that are canonical players within the immune system, including cytokines like interleukin (IL)‐6 and tumour necrosis factor alpha (TNFα), which are elevated during obesity, and contribute to inflammation [11]. In addition to these pro‐inflammatory cytokines, white adipocytes also express anti‐inflammatory IL‐10 [12, 13] suggesting that they can contribute to playing a role in regulating the immune response, as well as activating it (Fig. 1A). Based on emerging evidence discussed throughout this Viewpoint, we propose that adipocytes are immune hubs that integrate innate immune signals, using them to coordinate local and systemic responses to infection.

## The immune system and adipose tissue physiology

Under homeostasis, the adipose tissue is enriched with a multitude of immune cells, including monocytes, macrophages, dendritic cells, mast cells, eosinophils, B cells, CD4^+^ T cells, regulatory T cells (Tregs), CD8^+^ T cells, innate lymphoid cells (ILCs) and gamma delta (γδ) T cells [14, 15, 16, 17, 18, 19, 20, 21] (Fig. 1B). It is likely that under homeostasis and varying nutritional states, inflammatory and immunoregulatory signals from adipose tissue immune populations form a critical role in WAT remodelling. Beyond the adipose tissue, these resident immune populations can influence systemic metabolism. For example, IL‐33 signalling through adipose‐resident Tregs improves systemic glucose tolerance during obesity [22]. Further to this, IL‐17A^+^ γδ T cells drive IL‐33 expression from adipose stromal cells (including preadipocytes), which is critical for the maintenance of body temperature [23]. IL‐17A was defined as a key factor for maintaining body temperature, promoting thermogenic responses and driving adipose tissue expression of adipocyte genes associated with thermogenesis, including *Ucp1*. Indeed, the effect of IL‐17A on body temperature was profound, with cold exposure proving lethal for IL‐17A knockout mice. An independent study confirmed that IL‐17‐expressing γδ T cells are critical for thermogenesis and also identified that the effects are mediated through the adipocyte IL‐17 receptor C (IL‐17RC) [24]. IL‐17F signalling through IL‐17RC upregulated transforming growth factor β1 (TGF‐β1), which in turn supported adipose tissue innervation and thermogenesis. Numerous studies have explored the effects of other immune factors on adipose tissue thermogenesis, revealing that a range of different immune cells, including anti‐inflammatory macrophages [25] and type 2 innate lymphoid cells (ILC2s) [26, 27], contribute to this process.

The WAT has also been extensively studied in the context of obesity, where it has been shown that pro‐inflammatory cytokines such as TNFα signal directly to adipocytes, leading to adipocyte insulin resistance [28, 29]. This, in turn, elevates adipocyte lipolysis and export of free fatty acids (FFAs) into the circulation [30] and contributes to ectopic lipid deposition. TNFα was also found to be expressed by adipocytes themselves, acting in both an autocrine and paracrine manner [31, 32]. Numerous other cytokines (e.g. IL‐1β, IL‐6, IL‐17) have also been demonstrated to drive lipolysis in cultured adipocytes [33, 34, 35, 36], which is puzzling, as these cytokines are all elevated during obesity. In response to obesity, adipocytes also alter their transcriptional program to express immune‐related factors, including the chemokines *Ccl6*, *Ccl8*, *Ccl9* and *Cxcl12* [2, 37]. In the context of obesity in both humans and mice, the expression of adipocyte chemotactic networks promotes the recruitment of inflammatory myeloid cells, such as macrophages, further contributing to adipose tissue inflammation [38, 39]. Altogether, these studies demonstrate that the WAT is a rich immunological niche, and that adipocytes themselves are capable of producing factors that contribute to the maintenance of tissue homeostasis.

## Adipocyte lipolysis: responses to infection

Many studies of the adipose tissue have focused on understanding how it functions under homeostasis or how it is affected by obesity. These studies have provided essential insights into how adipocytes function and also respond/contribute to inflammation, but there is increasing interest in the response of the WAT to infection, and how it contributes to the ensuing immune response (Table 1). This is important from the point of view of understanding how the host fuels its immune response, but also for understanding whether adipocytes themselves contribute to that response.

**Table 1:** Pathogens that influence adipocyte function.

Pathogen	Disease	Effect on adipocytes	Host/model	Tissue type or cell line	Reference
**Viral**					
HIV	HIV and AIDS	Decreased lipolysis and fatty acid oxidation	Human	Serum	[56]
		Impaired adipogenesis	*In vitro—*mouse	3T3‐L1‐derived adipocytes	[114]
		Downregulation of PPARγ expression			
		Downregulation of adiponectin and glucose transporters	*In vitro*—mouse	SGBS adipocytes	[115]
		Upregulation of IL‐6, IL‐8 and MCP‐1			
Influenza A	Flu	Beiging of white adipose tissue (upregulation of *Ucp1*)	Mouse	Inguinal and gonadal adipose tissue	[40]
		Induction of a brown‐like phenotype in adipocytes	*In vitro*—mouse	3T3‐L1‐derived adipocytes	
		Upregulation of MCP‐1, CXCL10 and IFNγ	*In vitro*—human	Adipocytes differentiated from primary human preadipocytes (depot not described)	[116]
SARS‐CoV‐2	COVID	Decreased lipolysis	*In vitro—*human	Adipocytes derived from primary human subcutaneous preadipocytes	[46]
		Upregulation of inflammatory genes IFNA1, IFNB1, IER3, ISG15 and IFI27	*In vitro*—human	Adipocytes derived from primary human subcutaneous, visceral, pericardial or epicardial preadipocytes	[117]
		Upregulation of adiponectin, leptin and resistin in humans with acute respiratory distress syndrome	Human	Serum	[118]
			Syrian hamsters	Inguinal and gonadal adipose tissue	
		Downregulation of adiponectin and leptin	Mouse	3T3‐L1‐derived adipocytes	
		Elevated circulating adiponectin, leptin, resistin and visfatin	Human	Serum	[119]
**Bacterial**					
*Mycobacterium tuberculosis*	Tuberculosis	Upregulation of TNFα, IL‐6 and MCP‐1	*In vitro*—mouse	Mature adipocytes from inguinal adipose tissue	[120]
		Decreased adipocyte lipid droplet volume	Mouse	Gonadal adipose tissue	[121]
		Downregulation of adiponectin and PPARγ			
		Increased phosphorylation of hormone‐sensitive lipase			
		Elevated production of nitric oxide, TNFα, IL‐12p40, IL‐6, IL‐17 and IL‐4	*In vitro*—mouse	3T3‐L1 cells	[122]
		Increased lipid droplet volume (whitening effect)	Mouse	Brown adipose tissue	[123]
*Listeria monocytogenes*	Listeriosis	Transient increase in adipose tissue mass	Mouse	Perinodal adipose tissue	[124]
		Upregulation of adipocyte inflammatory cytokines (including *Il6*, *Cd86*, *Cd80*) and cytokine receptors (including *Ifnar1* and *Ifnar2*)		Isolated adipocytes from perinodal adipose tissue	
		Upregulation of *Nos2* driven by IFNγ	*In vitro*—mouse	3T3‐L1 cells and adipocytes derived from primary murine inguinal preadipocytes	
		Adipocytes engage the argininosuccinate shunt to generate nitric oxide and clear intracellular bacterial			
*Coxiella burnetii*	Q fever	Upregulation of inflammatory cytokines (e.g. *Il6*), chemokines (e.g. *Cxcl5*) and toll‐like receptors (e.g. *Tlr2*)	*In vitro*—mouse	3T3‐L1 cells	[125]
*Staphylococcus aureus*	Sepsis, skin infections, pneumonia	Upregulation of IL‐6, MCP‐1 and visfatin	*In vitro*—mouse	3T3‐L1 cells	[126]
		Downregulation of resistin and adiponectin			
		Inhibition of lipolysis			
		Upregulation of visfatin and leptin	Rat	Serum	[127]
		Downregulation of adiponectin		Serum	
		Upregulation of resistin and downregulation of AdipoR1		Gonadal adipose tissue	
		Downregulation of resistin and leptin	Mouse	Gonadal adipose tissue	
		Upregulation of visfatin		Serum	
*Chlamydia pneumoniae*	Pneumonia	Decreased adipose tissue mass	Mouse	Gonadal adipose tissue	[128]
		Decreased adipocyte lipid droplet volume			
		Elevated phosphorylation of hormone‐sensitive lipase			
		Elevated lipolysis and FABP4 secretion	*In vitro*—mouse	3T3‐L1 cells	[128, 129]
		Induces endoplasmic reticulum stress, activation of the unfolding protein response and reactive oxygen species production	*In vitro*—mouse	3T3‐L1 cells	[129]
**Parasitic**					
*Trypanosoma brucei*	Human African trypanosomiasis and African Animal trypanosomiasis	Decreased adipose tissue mass	Mouse	Gonadal, inguinal and brown adipose tissue	[47, 55]
		Decreased adipocyte lipid droplet volume	Mouse	Inguinal adipose tissue	[13, 47]
		Increased lipolysis during acute infection	Mouse	Gonadal and inguinal adipose tissue explants	[130, 131]
		Decreased lipolysis during chronic infection	Mouse, Human	Serum	[47]
		Promotes expression of pro‐ and anti‐inflammatory cytokines by adipocytes (*Tnf* and *Il10*, respectively)	Mouse	Subcutaneous adipocytes	[13]
		IL‐17 signalling through adipocytes partially controls local parasite numbers	Mouse	Inguinal adipose tissue	[47]
*Trypanosoma cruzi*	Chagas disease	Decreased adipose tissue mass and adipocyte lipid droplet volume	Mouse	White adipose (depot not described)	[62]
				Gonadal white adipose tissue	[132]
		Decreased circulating leptin and transient decrease in circulating adiponectin during chronic infection	Mouse	Serum	[132, 133]
		Downregulation of adiponectin, ATGL, HSL, pHSL, PPARγ, leptin, HSL, ATGL, FAS, LPL, DGAT1 and DGAT2	Mouse	Gonadal adipose tissue	[134]
				Mature adipocytes purified from gonadal adipose tissue	[132]
		Upregulation of PPARγ and downregulation of adiponectin		White adipose (depot not described) and brown adipose tissue	[58]
		Upregulation of cytokines (IL‐1β, IL‐4, IL‐10 and IFNγ and TNFα), chemokines (CCL2, CCL3, CCL5 and CXCL10), and toll‐like receptors (TLR2 and TLR9)	*In vitro*—mouse	3T3‐L1 cells	[62]
		Downregulation of PPARγ and adiponectin	*In vitro*—mouse	3T3‐L1 cells	[62, 135]
*Toxoplasma gondii*	Toxoplasmosis	Decreased adipose tissue mass	Mouse	Gonadal and inguinal adipose tissue	[136]
		Development of adipose tissue fibrosis	Mouse	Gonadal adipose tissue	[137]
*Plasmodium falciparum*	Malaria	Upregulation of leptin triggered by mTORC1 activity	Mouse	Serum and perigonadal adipose tissue	[93]
		Adipocyte leptin production correlates with development of cerebral malaria and mortality	Human	Subcutaneous adipose tissue	[83]
*Leishmania major*	Leishmaniasis	Elevated circulating leptin	Mouse	Serum	[138]
**Polymicrobial**					
Caecal‐ligation and puncture model of sepsis	Sepsis	Decreased adipocyte lipid droplet volume	Mouse	Inguinal and gonadal adipose tissue	[139]
		Increased number of mitochondria in adipocytes			
		Beiging of white adipose tissue (upregulation of Ucp1)			
		Increased nitric oxide and reactive oxygen species production	Mouse	Perivascular adipose tissue	[140]

Ayari *et al*. [40] identified that during influenza A infection (IAV), the WAT downregulates genes associated with lipolysis and lipogenesis, which appears to be counterintuitive, since lipolysis could potentially liberate nutrients that immune cells can use to function. For example, the induction of lipolysis and release of FFAs through pharmacological intervention or overnight fasting leads to an increase in adipose tissue macrophages [41, 42]. Additionally, cells such as T cells [43], germinal centre B cells [44] and anti‐inflammatory macrophages [45] all utilise free fatty acids to function. Despite this dichotomy, many studies have reported downregulation of lipolysis in the adipose tissue during different infection models, including during viral (SARS‐CoV‐2 [46]) and parasitic (*Trypanosoma brucei* [47]) infections, which was coupled with downregulation of adipose triglyceride lipase (ATGL) and hormone‐sensitive lipase (HSL), two enzymes that are critical for lipolysis (Table 1). Furthermore, whilst sepsis and endotoxemia are typically associated with elevation of lipolysis, in aged mice this effect is reversed, with B cell accumulation inhibiting lipolysis in the adipose tissue [48]. On the one hand, it is possible that the release of FFAs could be beneficial for resolving infection by promoting a more robust immune response. Conversely, it is possible to have too much of a good thing, and the release of FFAs may be lowered during infection to prevent inappropriate levels of immune cell activation and exacerbation of inflammation. In the case of SARS‐CoV‐2, it was demonstrated that stimulating lipolysis increased viral replication, leading to the hypothesis that anti‐lipolytic responses to infection represent part of an anti‐viral immune response. This is supported from studies of both IAV and SARS‐CoV‐2, where pharmacological inhibition of lipid droplet‐associated lipases conferred protection from severe infection and mortality [49]. The lipolytic response to *T. brucei* infection is interesting as lipolysis increases during the early stages of infection but decreases as the infection progresses to the chronic phase. Early activation of lipolysis may be important for supporting the innate immune response, including the recruitment of cells such as neutrophils [50, 51]. Moreover, lipolysis is also important for the function of adaptive immune cells, such as B cells, which use FFAs to support the production of cytokines such as IL‐10 [52], which is highly upregulated during *T. brucei* infection [53, 54]. Decreases in lipolysis that occur during chronic *T. brucei* infection may also represent a mechanism to slow replication of the parasite, as it has been shown that this parasite can take advantage of the lipid‐rich WAT environment, and upon colonisation of the tissue upregulates fatty acid oxidation to utilise the products of host lipolysis [55]. However, the impact of decreased lipolysis on the immune response to chronic infection remains unexplored.

Conversely, several studies have demonstrated the opposite effect, whereby infections can increase rates of adipocyte lipolysis, including viral (e.g. HIV [56]), bacterial (e.g. sepsis [57]) and parasitic (e.g. Chagas disease [58]) (Table 1). In the case of sepsis, early studies described that adipocytes sense bacterial endotoxins (LPS) via toll‐like receptor 4 (TLR4), which in turn stimulates lipolysis [59]. This raises the possibility that bacteria are able to stimulate lipolysis, driving the release of nutrients that they can use for their own survival and proliferation. However, more recent studies suggest that TLR4 is involved in mediating adipocyte‐immune crosstalk during bacterial infection, but that it does not necessarily drive lipolysis directly [60]. This may indicate that increased lipolysis during sepsis is driven by the immune response rather than the pathogen and supports the work from Wang *et al*. [61], demonstrating that fasting, which elevates lipolysis and release of FFAs, is protective during sepsis. During Chagas disease, which is caused by the intracellular *Trypanosoma cruzi* parasite, there is evidence that lipolysis also increases, and this is associated with upregulation of TLR2, TLR4 and TLR9 [58, 62]. However, *T. cruzi*‐derived molecules that could stimulate adipocyte TLR signalling have yet to be described, meaning that it remains unclear how infection with this intracellular parasite drives lipolysis. As with sepsis, it is possible that lipolysis is driven primarily by the immune response to the infection. If the modulation of lipolysis observed in different infections is driven by the immune response, then the question remains of why some immune responses induce lipolysis and others suppress it, and how does the host decide whether more or fewer resulting FFAs are required. Since FFAs are used by a number of immune cell types to fuel their activation, the answer to this question is likely to lie in understanding the precise nature of the immune response to the infection that the host is experiencing, in terms of the cell types that are recruited and the immune factors, such as cytokines and antibodies, that they make.

## Adipocytes as immune hubs: integration and dissemination of instructions

Both innate and adaptive immune responses to infection stimulate the release of an extensive range of cytokines and chemokines, the composition of which depends on the pathogen, the location of the infection, infection intensity and whether the infection is acute or chronic. There is a growing appreciation that adipocytes are not only capable of sensing these molecules but also responding to them and releasing a range of different factors that can contribute to the immune response. Probably, the most well‐described immune‐adipocyte interaction is between immune cell‐derived TNFα and adipocytes, which has long been known to drive lipolysis [63, 64, 65, 66]. However, it is now known that TNFα signalling also upregulates expression of a number of different cytokines and chemokines in adipocytes, including IL‐1β [67, 68], which is a key player in the antiviral response and is a pyrogen, acting on the brain to induce fever [69]. Moreover, white adipocytes secrete prostaglandin E2, a pyrogenic mediator that acts on the preoptic area of the brain and stimulates sympathetic nerve activity and brown adipose tissue (BAT) thermogenesis [70, 71], further supporting the role of WAT in mediating the response to infection.

Intriguingly, IAV drives beiging of scWAT, through upregulation of the mitochondrial biogenesis program, conferring a thermogenic phenotype during infection [40], which is remarkable considering that this infection is localised to the lungs but drives profound effects in the distal adipose tissue. This suggests that WAT beiging and downstream induction of thermogenesis form components of the antiviral response to infection, contributing to fever and disease resolution. TNFα was also found to stimulate leptin expression in the gonadal WAT (gWAT) of mice [72], an adipokine that suppresses hunger over time by signalling through the leptin receptor in neurons of the lateral hypothalamus [73]. In contrast, studies of the scWAT found that exposure of adipocytes to TNFα in this depot reduced tissue expression of leptin [74, 75], suggesting that there is heterogeneity in how adipocytes sense and respond to immune signals. Food intake is commonly suppressed (sickness‐induced anorexia) during infection and forms part of a behavioural program that is conserved across vertebrates and invertebrates [47, 76, 77], termed ‘sickness behaviour’. Therefore, it is likely that during infection, TNFα contributes to sickness‐induced anorexia by signalling through adipocytes to modulate adipokine expression and alter feeding behaviour. However, recent evidence from experimental *Toxoplasma gondii* infections suggests that this process may, in some scenarios, be driven independently of TNFα, via growth/differentiation factor 15 (GDF15) [78]. GDF15 is secreted by a range of tissues, including the liver, inguinal WAT and BAT [79], and can contribute to sickness‐induced anorexia independently of TNFα, via direct signalling in the hindbrain [80]. In *T. gondii* infection, GDF15 expression is driven by IFNγ and is critical for the sickness‐induced anorexia and weight loss associated with this disease [78]. Although much research has focused on the role TNFα as a driver of sickness behaviour and subsequent weight loss, it is becoming increasingly clear that a range of cytokines and adipokines, including IL‐1b, IL‐17, IL‐6 and leptin, contribute to these processes [47, 81, 82, 83], likely acting in tandem.

Beyond sickness‐induced anorexia, modulating leptin levels is critical for the immune response to numerous infections, as this adipokine signals through multiple different immune cell types to modulate their function, including macrophages [84], NK cells [85], neutrophils [86] and CD4^+^ T cells (specifically IL‐17^+^ T_H_17 cells [87]). However, the role of leptin in the immune response to infection is not straightforward, and it can either be beneficial or harmful depending upon the nature of the infection. For example, during *Leishmania donovani* [88], *T. cruzi* [89], *Mycobacterium tuberculosis* [90, 91] and *Pneumococcal pneumonia* [92] infections, elevated leptin is beneficial for improving the immune response, clearing pathogens and resolving infection. Conversely, in the context of infections like malaria, sequestration of infected red blood cells in the WAT drives leptin expression, which drives the development of cerebral malaria, increasing the risk of death [83, 93].

**Fig. 2:**
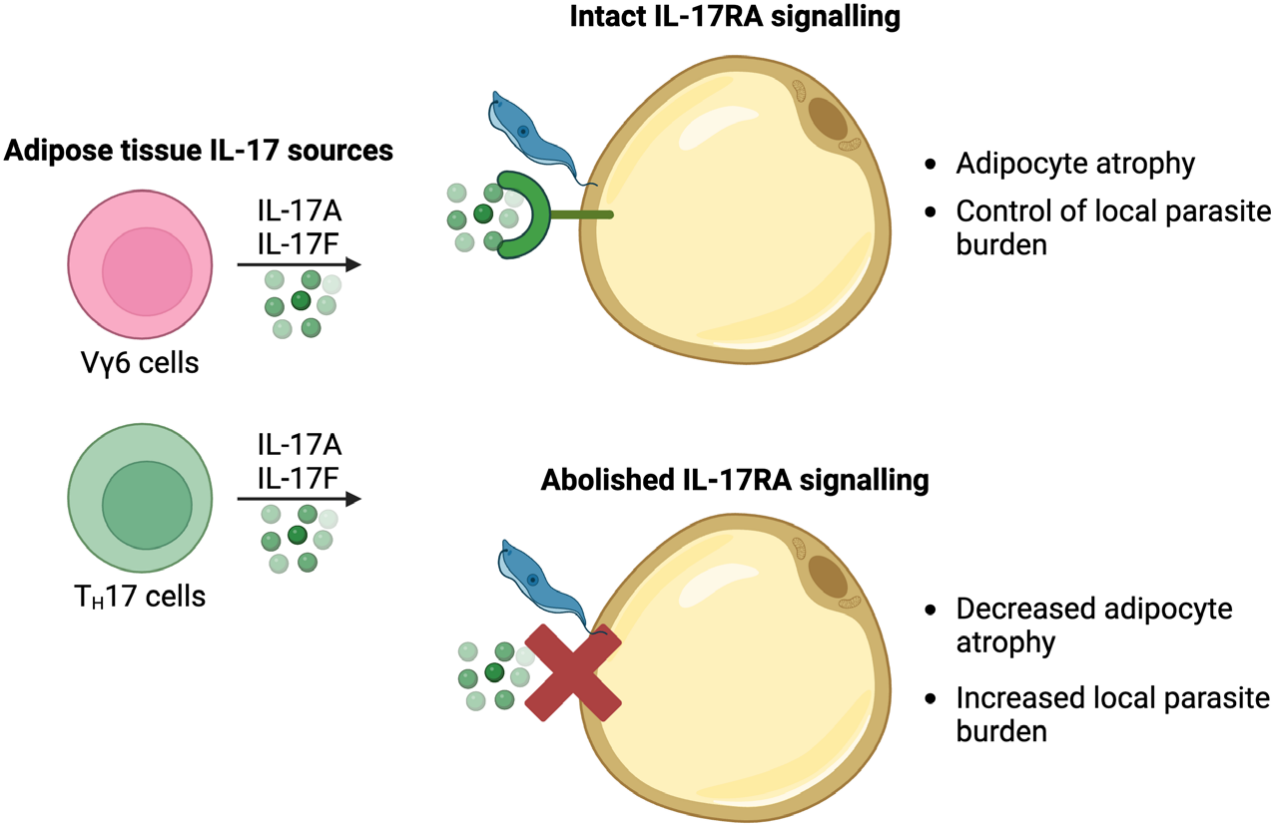
Effects of IL‐17 receptor A signalling during infection. In the inguinal white adipose tissue (iWAT), there are multiple sources of IL‐17A and IL‐17F, including Vγ6 and T_H_17 cells. We previously identified that these cytokines signal through the adipocytes during *Trypanosoma brucei* infection to drive adipocyte atrophy. When IL‐17 receptor A was deleted from adipocytes, they experienced less atrophy during infection, demonstrating that IL‐17 acts directly on the adipocytes to drive this process. Moreover, we found a higher number of parasites in the iWAT when adipocytes were unable to sense IL‐17, indicating that the adipocytes are able to integrate local immune signals and either directly affect *T. brucei* or disseminate these signals to the local immune system, and contributing to an environment that is more conducive for mounting an effective immune response.

As discussed throughout this Viewpoint, many other cytokines act upon adipocytes and stimulate lipolysis, or the release of cytokines and adipokines that have effects on the immune system during infection. In recent studies, we identified that IL‐17 signalling through adipocytes during infection with *T. brucei* limited the characteristic adipocyte atrophy associated with this disease, which occurred in a sex‐dependent manner [13, 47]. During *T. brucei* infection, in addition to increased secretion of IL‐17A by CD4^+^ and γδ T cells, adipocytes upregulate the cognate receptor, IL‐17 receptor A (IL‐17RA) [13] and IL‐17 receptor C (IL‐17RC) [47]. Whilst IL‐17 signalling is known to play a role in modulating adipose tissue structure and function during obesity [94], as well as modulating functions such as thermogenesis [23, 24], it remains unclear whether this is due to upregulation of the cognate IL‐17 receptors, or increased signalling through them. Strikingly, when we deleted adipocyte IL‐17RA, limiting the ability of adipocytes to sense IL‐17A and IL‐17F, there was a significant increase in the number of parasites in the scWAT [47] (Fig. 2). This unexpected observation suggests that IL‐17 signalling through adipocyte IL‐17RA primes the adipocytes to either directly control local parasite burden, perhaps through the release of antimicrobial peptides, such as cathelicidin [95]. In mouse models of *T. brucei* infection, cathelicidins were found to decrease parasite viability and delay infection‐induced mortality [96], but it has not yet been demonstrated whether adipocytes express these trypanolytic compounds during infection. However, as adipocytes secrete cathelicidins, alongside other antimicrobial compounds, such as lipocalins [97], this could potentially represent a direct way in which adipocytes are able to control local pathogen numbers. It is also possible that IL‐17 signalling, by driving adipose tissue wasting and adipocyte atrophy, induces the release of specific lipid species, such as sphingolipids, which have antimicrobial properties [98], or can act as signalling molecules to modulate the local immune response [99]. In this regard, lipids such as fatty acids have been demonstrated to influence both differentiation and activation of immune cells, including CD8^+^ T cells [100], and macrophages [101]. Alternatively, these findings may suggest that IL‐17 signalling in adipocytes promotes the expression of different cytokines and chemokines, to orchestrate the local immune response and control the pathogen numbers in the WAT. Adipocytes are also a major source of complement proteins, including C3, which is capable of killing African trypanosomes prior to them engaging mechanisms to evade immune attacks, namely antigenic variation [102]. As IL‐17 can promote expression of C3 in fibroblasts, it is possible that IL‐17 signalling through adipocytes helps to control parasite numbers by modulating the complement system. Whilst the exact mechanism underpinning this effect remains unknown, and whether this is a direct or indirect effect, it demonstrates that the adipocytes of the scWAT are able to sense immune signals, integrate them and then exert a profound effect on the outcome of infection. Furthermore, it will be important to understand whether sex plays a role in mediating the effects of IL‐17 on adipocytes and their downstream immune effector function. We observed that IL‐17 drives weight loss during *T. brucei* infection in males but not in females, and previous studies of urinary tract infection found that IL‐17 was critical for resolving infection in females but not males [103]. Together, this demonstrates that there are many factors influencing the role that IL‐17 plays during infection, and there is still much to be uncovered.

## Conclusions and future perspectives

Whilst there are many questions left to answer about the response of adipose tissue to infection, there are also technological challenges that need to be addressed. Adipose tissue is notoriously challenging to work with for multiple reasons, including the loose structure of the tissue, the fragility of the adipocytes themselves and the abundance of lipids within the tissue. Single‐nucleus RNA sequencing (snRNAseq) is increasingly being used to interrogate the response of the adipose tissue to different challenges. An issue facing snRNAseq is that it does not capture cytosolic mRNA molecules, but recent advances have been made to enable the capture of the endoplasmic reticulum in addition to the nucleus, maximising mRNA capture [3, 104]. Another issue is that adipocytes are rich in RNAseq, leading to extensive degradation of mRNA. So *et al*. [105] have now demonstrated that using protease inhibitors, as well as RNAseq inhibitors, overcomes this obstacle and preserves mRNA integrity during nuclei isolation. In tandem spatial transcriptomics [4, 13, 106] and emerging single‐cell proteomics [107], snRNAseq will undoubtedly advance our understanding of how adipose tissue functions and responds to challenges. These technologies will also benefit from the range of genetic deletion models that are now available to the adipocyte biology field, including mice with constitutive or inducible deletions of genes in white (*Adipoq*‐Cre, *Adipoq*‐CreERT2 [108, 109]) and brown (*Ucp1*‐Cre, *Ucp1*‐CreERT2 [110, 111]) adipose tissue. Although these markers are useful for studying mature adipocytes, a major challenge in the field is identifying genes that are specific to adipocyte precursor cells, and overcoming this will enable deeper studies of adipocyte development and plasticity. Recent studies have used lineage tracing to overcome the lack of deletion models, elegantly demonstrating the trajectory of preadipocytes to a white [112] or beige adipocyte lineage [113]. These technological advances have already improved our understanding of adipocyte biology in mice and humans, and as they continue to develop will allow the field to develop deeper insights into how adipocytes respond to challenges such as obesity and infection, and how they contribute to the immune response.

We have learned a lot about the immune responses of adipocytes from studies of obesity, but obesity is a relatively modern disease, and it is likely that adipocytes evolved to express immune factors under different evolutionary pressures, such as exposure to injury and infection. Under these conditions, it is emerging that adipocytes are key contributors to the immune response, but we still know little about adipose depot‐specific responses to infection, and whether these responses are impaired when there is an excess of adipose tissue, such as during obesity. Furthermore, we do not yet understand how local infections, such as in the lung, are able to trigger distal responses in the adipose tissue, or what the benefit of this is to the host. One possibility is that an infection in the lung could prompt the adipose tissue to release FFAs and adipokines into circulation, which can then be used to mount an appropriate immune response. Understanding these interactions may be important for a clinical point of view, where a patient is underweight or obese and has an impaired adipose tissue response.

We propose that during infection the adipocyte forms a cellular hub that integrates incoming immune signals and uses them to coordinate the local and systemic immune response, in turn making the adipose tissue a key, but underappreciated, player in the immune system.

## Conflict of interest

The authors declare no conflict of interest.

## Author contributions

MCS and SK conceptualised the manuscript. MCS wrote the manuscript.
